# Probiotics Can Boost the Antitumor Immunity of CD8^+^T Cells in BALB/c Mice and Patients with Colorectal Carcinoma

**DOI:** 10.1155/2020/4092472

**Published:** 2020-05-09

**Authors:** Jie Mao, Shu-Ze Zhang, Peng Du, Zhi-Bin Cheng, Huan Hu, Shi-Yao Wang

**Affiliations:** ^1^The Department of General Surgery, Lanzhou University Second Hospital, Lanzhou, 730030 Gansu, China; ^2^Second Clinical Medical College, Lanzhou University, Lanzhou, 730030 Gansu, China

## Abstract

**Background:**

The drug resistance and the immune suppression in the tumor microenvironment are important factors affecting tumor progression. Reversing drug resistance and changing tumor suppression microenvironment are ideal ways to inhibit tumor progression.

**Objective:**

The aim of the study is to verify antitumor immune response of probiotics in patients with colorectal carcinoma and to explore its mechanism.

**Methods:**

To detect the tumor samples of 122 patients with colorectal carcinoma after surgery, analyze the effect of probiotics on enhancing tumor-infiltrating CD8^+^T cells to inhibit colorectal carcinoma, and further verify the mechanism of probiotics on enhancing the antitumor immune response of CD8^+^T cells through animal experiments.

**Results:**

The results of immunohistochemistry showed that the proportion of CD8^+^T cells in the patients treated with probiotics before surgery was increased significantly than that in other patients (*P* = 0.033). The results of flow cytometry also showed that the proportion of CD8^+^T cells in the probiotics group was higher than that in the nonprobiotics group (*P* = 0.029). Kaplan-Meier survival estimates also showed that the CD8^+^T cells, TNM stage, pathology grade, lymphatic metastasis, and probiotic treatment were significantly associated with the progression-free survival (PFS) (*χ*^2^ = 9.684, *P* = 0.002 for CD8^+^T cells; *χ*^2^ = 5.878, *P* = 0.015 for TNM stage; *χ*^2^ = 7.398, *P* = 0.004 for pathology grade; *χ*^2^ = 8.847, *P* = 0.003 for Lymphatic metastasis; and *χ*^2^ = 4.622, *P* = 0.032 for the group (group A was treated with probiotics before surgery; group B was not treated with probiotics)). The experimental results in mice showed that probiotics could inhibit tumor growth and increase the proportion of CD8^+^T cells in mice; the difference was statistically significant (*P* = 0.037). It was also found that probiotic feeding could upregulate the expression of T-cell immunoglobulin mucin receptor 1(TIM-1) in CD8^+^T cells of mice and also found that probiotic feeding could downregulate the expression of programmed cell death protein 1 (PD-1) in CD8^+^T cells of mice, compared with the nonfeeding group; the difference was statistically significant (*P* = 0.045 for TIM-1 and *P* = 0.02 for PD-1, respectively). In order to further understand the functional status of CD8^+^T cells, we analyzed interferon-gamma (IFN-*γ*)^+^ T cells and tumor necrosis factor-*α* (TNF-*α*)^+^CD8^+^T cells by flow cytometry. The results showed that the proportion of IFN-*γ*^+^ T cells and TNF-*α*^+^CD8^+^T cells significantly increased after probiotic treatment, compared with the nonprobiotic treatment group; the difference was statistically significant (*P* = 0.040 for IFN-*γ*^+^ T cells and *P* = 0.014 for TNF-*α*^+^CD8^+^T, respectively).

**Conclusions:**

Probiotics can enhance the antitumor immune response of CD8^+^T cells. It can play a synergistic antitumor role. On the one hand, its mechanism is through regulating intestinal flora, and on the other hand, through regulating the antitumor immune function of CD8^+^T cells.

## 1. Introduction

Colorectal carcinoma is a common malignant tumor of the digestive system [1, 2]. Surgery is an important method of its treatment; chemotherapy after surgery is the key to reduce the recurrence and metastasis of tumor. However, some patients who receive standard chemotherapy after surgery will still have recurrence and metastasis. This is mainly due to the immune suppression in the tumor microenvironment of the human body to tumor and the drug resistance of tumor to chemotherapy. Probiotics are important intestinal flora regulators, which are mainly used for the treatment of intestinal flora disorders. Recent research results show that probiotics such as bifidobacteria can reduce the drug resistance of tumor to programmed cell death protein 1 (PD-1) and cytotoxic T-lymphocyte-associated protein 4 (CTLA-4) molecular-targeted drugs, improve its efficacy, and increase the number of CD8^+^T cells. Bifidobacteria can play a synergistic antitumor effect by combining with PD-1 and CTLA-4 molecular-targeted drugs. By regulating the intestinal flora of the human body, it can reduce the incidence of drug resistance of tumor to molecular-targeted drugs. In addition, it can also change the immunosuppressive state of patients with tumor and stimulate antitumor effect [3–5].

At present, probiotics, such as Bifidobacterium capsules, have been widely used in clinical treatment of diseases related to flora disorders, which also affect human immune function and nutritional metabolism [6–8]. Recent study results show that probiotics can enhance the antitumor immunity and improve the therapeutic effect of tumor through its immunoregulation [7, 9–11]. It has also been found that the mechanism of tumor resistance is mainly related to some bacteria in patients, such as cytidine deaminase which is produced by Proteus entericus, which can make gemcitabine inactive, thus weakening or disappearing its antitumor effect [3]. It has also been shown that intestinal microorganisms can play an antitumor role by regulating innate immunity and acquired immunity [12], and it has also been found that Bifidobacterium can increase the antitumor effect of CD8^+^T cells in tumor tissue through dendritic cells and enhance the antitumor effect of PD-1 and CTLA-4 molecular-targeted drugs [13].

Therefore, we propose that probiotics can not only regulate intestinal flora to maintain homeostasis but also reduce the occurrence of tumor resistance and play a synergistic antitumor effect through positive regulation of T cell-mediated immune response. In this study, the mechanism of probiotics (bifidobacteria) stimulating and enhancing the antitumor immune response of CD8^+^T cells was verified based on in vivo and in vitro experiments.

## 2. Materials and Methods

### 2.1. Patients

#### 2.1.1. Clinical Data

We enrolled 122 patients with colorectal carcinoma who underwent tumor resection at the Second Hospital of Lanzhou University (Gansu Province, China) between 2014 and 2017. The study was approved by the Ethics Committee of at the Second Hospital of Lanzhou University, and the data were anonymously obtained and analyzed. Informed consents from participants were also waived due to the complete anonymity of the patients. The data of all patients, including laboratory data and pathological data, were collected from the electronic medical records of the Second Hospital of Lanzhou University. This study was performed in accordance with the relevant guidelines and regulations and conformed to the Declaration of Helsinki [14]. The inclusion criteria of patients included complete pathological and follow-up data, without long distance metastasis, without chronic diseases, and without any treatments before the surgery. The exclusion criteria are patients who suffered from other tumor or other chronic diseases or died from accidental death or other diseases, lack of pathological and follow-up data, and long distance metastasis before the surgery. Ultimately, a total of 122 cases were eventually enrolled in this study, including 58 males and 64 females, aged 28 to 82 years, median age of 59 years.

### 2.2. Immunohistochemistry

Immunohistochemistry procedures and evaluation of formalin-fixed, paraffin-embedded tissues were processed for immunohistochemical staining with antibodies for CD4 (clone: SP35, Maixin Biotechnology Co.) and CD8 (clone: SP16, Maixin Biotechnology Co.). CD4^+^ T cells and CD8^+^T cells were classified as low if <25% of tumor cells were stained; otherwise, they were categorized as high. High was assorted further according to the levels (≥26%). During statistical analysis, it was assorted synoptically as either low or high.

### 2.3. Cell Lines and Cell Culture and Animal Models

The mouse colon cancer cell line CT26 was obtained from Chinese Academy of Sciences, Shanghai Institutes for Biological Sciences (Shanghai, China).1 × 10^6^ CT26 was symmetrically injected i.d. into male BALB/C mice.

### 2.4. Flow Cytometric Analysis

The tumor-infiltrating lymphocytes were harvested according to the method. In brief, the tumor masses were removed, minced, and digested with collagenase and hyaluronidase solution. The cell suspension was filtered through a cell mesh and resuspended in Hank's media plus 1% fetal calf serum (FCS) for further analysis. The main antibodies are CD4 (GK1.5), CD8 (53-6.7), IFN-*γ* (XMG1.2), TNF-*α* (MP6-XT22), PD-1 (RPM1-30), etc. These antibodies were purchased from BioLegend. Flow cytometric analysis was performed using a FACS flow cytometer (Becton Dickinson). For intracellular cytokine staining, harvested cells were stimulated with PMA (10 ng/ml) and ionomycin (1 *μ*g/ml) for 4 h and incubated for the last one hour with brefeldin A (10 *μ*g/ml). IFN-*γ*- and TNF-*α*-producing cells were examined with flow cytometry.

### 2.5. Statistical Analysis

SPSS 22.0 statistical software was used to analyze all data. Kaplan-Meier survival estimate model was used to assess the correlation of the progression survival (PFS) with gender, age, CD4^+^T cells, CD8^+^T cells, lymphatic metastasis, and chemotherapy regimen. *P* < 0.05 was considered statistically significant. The proportion difference of T cells after probiotic treatment was analyzed by independent sample *t*-test.

## 3. Results

### 3.1. The Results of Clinical Experiments and Statistical Analysis

**Figure 1:**
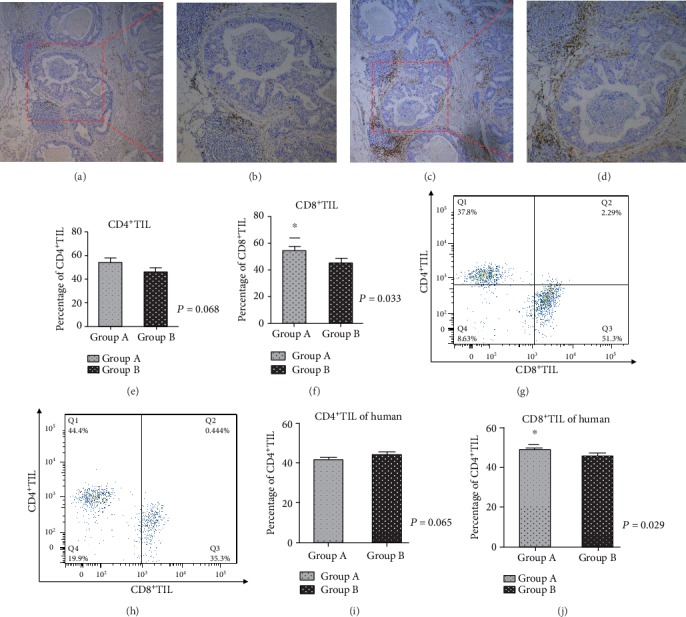
Immunohistochemical and flow cytometry results of human colorectal carcinoma. (a–d) The immunohistochemical results of consecutive sections of the same patient's colorectal cancer tissue; (a, b) the results of CD4^+^T cell staining; (c, d) the results of CD8^+^T cell staining. (e) The statistical figure of CD4^+^T cells between the probiotic treatment group and the nonprobiotic treatment group. (f) The statistical figure of CD8^+^T cells between the probiotic treatment group and the nonprobiotic treatment group. (g, h) Flow cytometric results of CD4^+^T cells and CD8^+^T cells. (i, j) Statistical results. Note: group A was treated with probiotics before surgery; group B was not treated with probiotics.

The CD4^+^T cells and CD8^+^T cells in 122 tissue samples from patients with colorectal carcinoma were detected by immunohistochemistry (Figures 1(a)–1(d)). We found that the proportion of CD8^+^T cells increased significantly in patients treated with probiotics before surgery. The results of independent sample *t*-test showed that the infiltration rate of CD4^+^T cells in the patients treated with probiotics increased, compared with that in the patients treated with nonprobiotics, but there was no statistical significance (*P* = 0.068, Figure 1(e)). The proportion of CD8^+^T cells in the patients treated with probiotics was significantly higher than that in the patients treated with nonprobiotics, and the difference was statistically significant (*P* = 0.033, Figure 1(f)). In addition, the tissue samples of colorectal carcinoma were detected by flow cytometry (Figures 1(g) and 1(h)), and the results were basically consistent with the immunohistochemical results. The results showed that there was no statistical significance in the change of CD4^+^T cells (*P* = 0.065, Figure 1(i)), but the proportion of CD8^+^T cells in the probiotic treatment group was higher than that in the nonprobiotic treatment group, and the difference was statistically significant (*P* = 0.029, Figure 1(j)). The following is a typical figure of experimental results.

### 3.2. Statistical Results of the Patients with Colorectal Carcinoma

**Figure 2:**
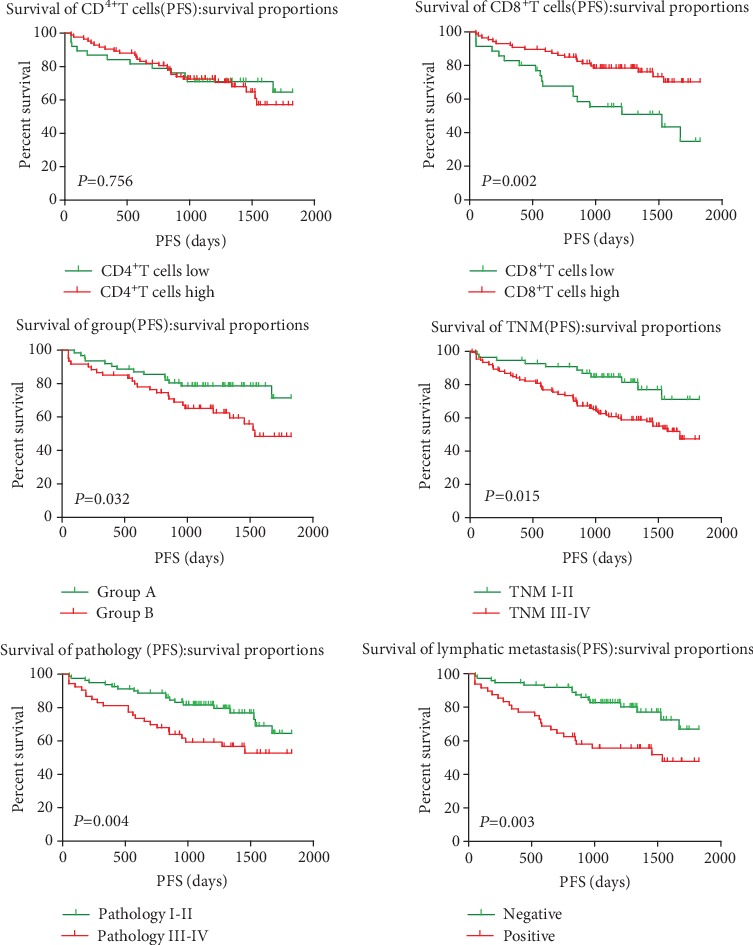
Clinical factors affecting the PFS of patients.

Results showed that the CD8^+^T cells, TNM stage, pathology grade, lymphatic metastasis, and probiotic treatment were significantly associated with the progression-free survival (PFS) (*χ*^2^ = 9.684, *P* = 0.002 for CD8^+^T cells; *χ*^2^ = 5.878, *P* = 0.015 for TNM stage; *χ*^2^ = 7.398, *P* = 0.004 for pathology grade; *χ*^2^ = 8.847, *P* = 0.003 for lymphatic metastasis; and *χ*^2^ = 4.622, *P* = 0.032 for the group (group A was treated with probiotics before surgery; group B was not treated with probiotics)), but it is not related to other clinical factors (Table 1, Figure 2).

### 3.3. The Results of Tumor Inhibition by the Colon Carcinoma Model of BALB/c Mice

**Figure 3:**
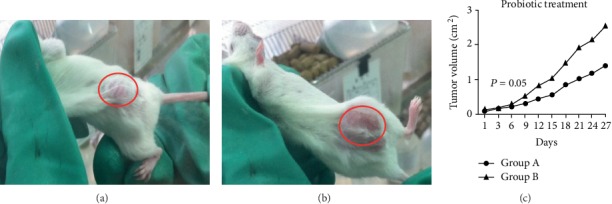
Tumor growth curve of mice after probiotic intervention. After CT26 cells were inoculated subcutaneously on the back of BALB/c mice, they were fed with bifidobacteria to measure the tumor growth state (a, b); the statistical diagram of tumor growth (c). Note: group A is the probiotic feeding group; group B is the nonprobiotic feeding group.

The mice of the research group were fed with Bifidobacterium preparation immediately after tumor formation, the growth of tumor was observed, the length (*a*) and width (*b*) of the tumor were measured with a vernier caliper, and the volume of the tumor is calculated according to the formula *V* = *a* × *b*^2^/2. It was observed that the tumor growth of the probiotic feeding group (group A) was slower than that of the nonprobiotic feeding group (group B), but there was no statistical difference (*P* = 0.05) (Figure 3).

### 3.4. The Analysis of the Mechanism of Tumor Inhibition by the Colon Carcinoma Model of BALB/c Mice

**Figure 4:**
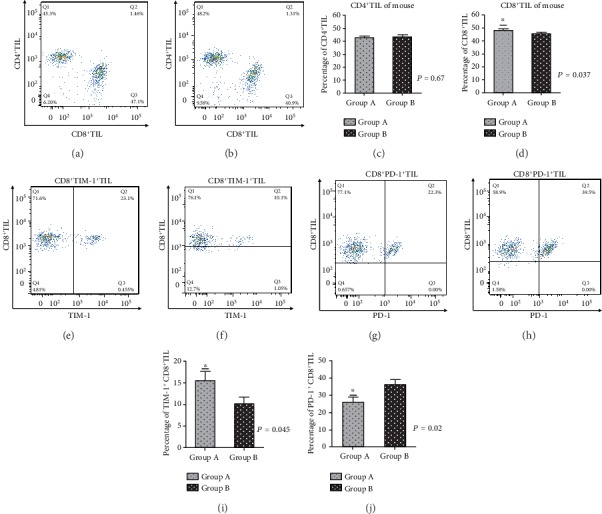
Analysis of antitumor mechanism of CD8^+^T cells after probiotic intervention. After CT26 cells were inoculated subcutaneously on the back of BALB/c mice, they were fed with Bifidobacterium. After 3 days, the tumor tissues were removed and analyzed by flow cytometry. (a) The results of CD4^+^TIL and CD8^+^TIL in the Bifidobacterium-fed mice, (b) the results of CD4^+^TIL and CD8^+^TIL in the non-Bifidobacterium-fed mice, (c) the statistical results of CD4^+^TIL between group A and group B, (d) the statistical results of CD8^+^TIL between group A and group B, (e) the results of CD8^+^TIM-1^+^TIL in the Bifidobacterium-fed mice, (f) CD8^+^TIM-1^+^TIL in mice fed with nonbifidobacteria, (g) CD8^+^PD-1^+^TIL in mice fed with bifidobacteria, (h) CD8^+^PD-1^+^TIL in mice fed with nonbifidobacteria, (i) statistical result of TIM-1 expression, and (j) statistical result of PD-1 expression. Note: group A is the probiotic feeding group; group B is the nonprobiotic feeding group.

**Figure 5:**
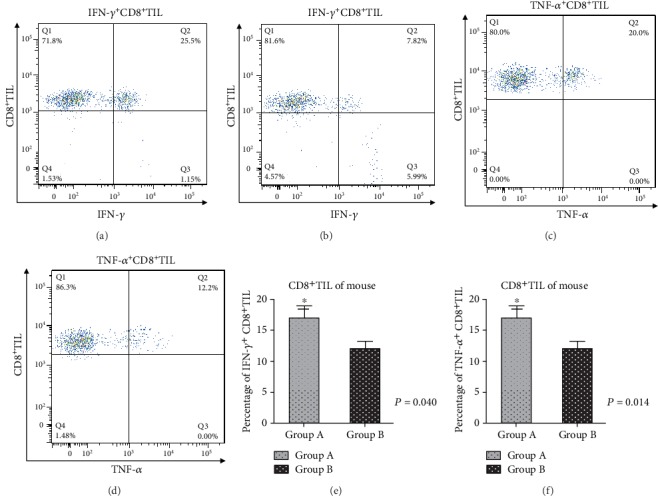
Analysis of antitumor mechanism of CD8^+^T cells after probiotic intervention. After CT26 cells were inoculated subcutaneously on the back of BALB/c mice, they were fed with Bifidobacterium. After 3 days, the tumor tissues were removed and analyzed by flow cytometry. (a) The results of IFN-*γ*^+^CD8^+^TIL in the Bifidobacterium feeding group, (b) the results of IFN-*γ*^+^CD8^+^TIL in the non-Bifidobacterium feeding group, (c) the TNF-*α*^+^CD8^+^TIL in the Bifidobacterium feeding group, (d) the results of TNF-*α*^+^CD8^+^TIL in the non-Bifidobacterium feeding group, (e) the statistical results of IFN-*γ*^+^CD8^+^T cells between group A and group B, and (f) the statistical results of TNF-*α*^+^CD8^+^T between group A and group B. Note: group A is the probiotic feeding group; group B is the nonprobiotic feeding group.

After the colon carcinoma model of BALB/c mice was constructed, the mice were fed with Bifidobacterium, and the tumor tissue was removed at 3 days and analyzed by flow cytometry. The results were basically the same as the tissue samples of human colon carcinoma. There was no significant difference of the CD4^+^T cells in the two groups (*P* = 0.67), but the proportion of CD8^+^T cells in mice fed with probiotics was upregulated. Compared with the nonprobiotic feeding group, the difference was statistically significant (*P* = 0.037). In order to understand the molecules that may be involved in immune regulation, the expression of T cell immunoglobulin mucin receptor 1 (TIM-1) costimulatory molecules in CD8^+^T cells was also detected and analyzed in this experiment. The results showed that probiotic feeding could upregulate the expression of T cell immunoglobulin mucin receptor 1 (TIM-1) in CD8^+^T cells of mice and also found that probiotic feeding could downregulate the expression of programmed cell death protein 1 (PD-1) in CD8^+^T cells of mice, compared with the nonfeeding group, the difference was statistically significant (*P* = 0.045 for TIM-1 and *P* = 0.02 for PD-1, respectively) (Figures 4(a), 4(b), and 4(e)–4(j)). In order to further understand the functional status of CD8^+^T cells, interferon-gamma (IFN-*γ*) and tumor necrosis factor-*α* (TNF-*α*)^+^CD8^+^T cells were analyzed by flow cytometry. The results showed that the proportion of IFN-*γ*^+^ and TNF-*α*^+^CD8^+^T cells significantly increased after probiotic treatment. Compared with the nonprobiotic treatment group, the difference was statistically significant (*P* = 0.040 for IFN-*γ*^+^CD8^+^T cells and *P* = 0.014 for TNF-*α*^+^CD8^+^T cells, respectively) (Figures 5(a)–5(f)). The following is a typical figure of experimental results.

## 4. Discussion

At present, probiotics, such as Bifidobacterium, have been widely used in clinical treatment of diseases related to flora disorders, which mainly affect human immune function and nutritional metabolism [6–8]. Recent research results showed that probiotics can enhance the antitumor immunity and improve the therapeutic effect of tumor through its immunoregulation [7, 9–11]. The research results published in Science showed that Bifidobacterium was used with programmed cell death protein 1 (PD-1) and cytotoxic T-lymphocyte-associated protein 4 (CTLA-4) molecular-targeted drugs together which can play a synergistic antitumor effect. It can reduce the incidence of tumor resistance to molecular-targeted drugs and improve its efficacy. In addition, it can also change the immunosuppressive state of patients with tumor and stimulate the antitumor effect [3–5]. In our previous clinical work, probiotics (bifidobacteria, etc.) have been given to patients undergoing FOLFOX chemotherapy for patients with gastric carcinoma or colorectal carcinoma. It has been found that probiotics have improved their appetite, digestive tract symptoms caused by chemotherapy, and whole body state, but whether it has an impact on tumor resistance and survival is still under further observation. The intestinal probiotics may not only improve the state of the body by changing the intestinal flora but also can reduce the resistance of tumor to FOLFOX chemotherapy drugs.

It has been found that the molecular mechanism of tumor resistance is mainly related to some bacteria in patients. For example, cytidine deaminase produced by Gammaproteobacteria can make gemcitabine inactive, thus weakening or disappearing its antitumor effect [3]. It has also been shown that intestinal microorganisms can play an antitumor role by regulating innate immunity and acquired immunity [12], and it has also been found that probiotic Bifidobacterium can increase the antitumor effect of CD8^+^T cells in tumor tissue through dendritic cells and enhance the antitumor effect of PD-1 and CTLA-4 molecular-targeted drugs [13]. Our study also showed that the proportion of CD8^+^T cells in tumor tissue of patients treated with probiotics was significantly higher than that of patients treated with nonprobiotics. The proportion of CD8^+^T cells in tumor tissue of mice fed with probiotics (Bifidobacterium) was significantly higher than that of mice fed with nonprobiotics, and it was also found that T cell immunoglobulin mucin-1 (TIM-1), a costimulatory molecule of T cell, was highly expressed, while PD-1 was downregulated, and interferon-gamma (IFN-*γ*)^+^CD8^+^TIL and tumor necrosis factor-*α* (TNF-*α*)^+^CD8^+^TIL were also increased. Studies have shown that TIM-1, like TLR4, is a very important T cell immune regulatory molecule, which plays an important role in mediating T cell immunity. It has been found that TIM-1 is related to antitumor immunity [15, 16]. Therefore, we speculate that probiotics, as an immunomodulator, may also enhance the antitumor immune response of T cells through the TIM-1 signaling pathway.

TIM-1 is a kind of molecule mainly expressed on the surface of T cells, which is closely related to the activation of immune cells. Tim family members are transmembrane glycoproteins with common motifs. TIM-1 is involved in TCR-mediated T cell activation [17, 18]. Up to now, it has been found that TIM-4 and phosphatidylserine (ptdser) are the two main ligands of TIM-1; TIM-4 is the endogenous ligand of TIM-1, which is expressed on the surface of activated antigen-presenting cells and mediates the positive regulation of T cell activation through TIM-1/TIM-4 ^[^19^]^; ptdser (PS) is another important ligand of TIM-1. As an important pattern recognition receptor on the membrane surface of NKT cells, TIM-1 can activate NKT cells by recognizing PS signals of apoptotic cells. The combination of these two ligands with TIM-1 can activate positive costimulatory signals [20, 21]. Recent studies also show that P-selectin and s-selectin are potential ligands of TIM-1 in inflammatory and autoimmune diseases, which are closely related to the migration of Th1 and Th17 cells in the blood vessels [22]. TIM-1 signal plays an important role in CD8^+^T cell function. Therefore, further study on the function and mechanism of costimulatory molecule TIM-1 regulating CD8^+^T cells is of great value to further clarify the synergistic antitumor effect and mechanism of probiotics.

Therefore, we propose the following hypothesis: on the one hand, probiotics mediate the antitumor immunity of CD8^+^T cells through the TIM-1 signal pathway; on the other hand, probiotics improve the state of the body and regulate it by changing the intestinal flora spectrum and the expressed enzyme spectrum. It can affect the expression of PD-1 and CTLA-4 that can reduce the incidence of drug resistance and play a synergistic antitumor role.

## Figures and Tables

**Table 1:** The correlation between clinical data and PFS of patients with colorectal carcinoma was analyzed by log-rank test.

Clinical parameters	Cases	Median PFS (days)	*χ* ^2^	*P*
Gender				
Male	63	1372	0.766	0.382
Female	59	1453		
Age				
<59	58	1334	2.023	0.155
≥60	64	1480		
CD4^+^T cells				
Low	38	1408	0.097	0.756
High	84	1412		
CD8^+^T cells				
Low	35	1514	9.684	0.002
High	87	1158		
Group				
Group A	62	1531	4.622	0.032
Group B	60	1294		
TNM stage				
I-II	55	1566	5.878	0.015
III-IV	67	1285		
Pathology grade				
I-II	80	1531	7.398	0.004
III-IV	42	1183		
Lymphatic metastasis				
Positive	48	1186	8.847	0.003
Negative	74	1558		

Note: group A was treated with probiotics before surgery, group B was not treated with probiotics.

## Data Availability

The clinical data used to support the findings of this study are included in the article.
